# Microbial Exopolysaccharides, Redox Modulation, and Antioxidant Activity in Fermented Foods

**DOI:** 10.3390/antiox15060665

**Published:** 2026-05-25

**Authors:** Fares Boudjouan, Giorgia Perpetuini, Rosanna Tofalo, Yves Waché, Nadjet Benaida Debbache

**Affiliations:** 1 Laboratoire de Génie de l’Environnement, Faculté de Technologie, Université de Bejaia, Bejaia 06000, Algeria; fares.boudjouan@univ-bejaia.dz; 2 Département de Biotechnologie, Faculté des Sciences de la Nature et de la Vie, Université de Bejaia, Bejaia 06000, Algeria; 3 Department of Bioscience and Technology for Food, Agriculture and Environment, University of Teramo, Via Balzarini 1, 4100 Teramo, Italy; gperpetuini@unite.it (G.P.); rtofalo@unite.it (R.T.); 4 Université Bourgogne Europe, Institut Agro, INRAE, UMR PAM, 21000 Dijon, France; 5 Laboratoire de Biochimie Appliquée, Faculté des Sciences de la Nature et de la Vie, Université de Bejaia, Bejaia 06000, Algeria

**Keywords:** fermented food, antioxidants, redox, lactic acid bacteria, exopolysaccharides

## Abstract

Oxidative stress, caused by the excessive production of reactive oxygen and nitrogen species, contributes to cellular damage and chronic diseases. Fermented foods are increasingly recognized for their antioxidant properties, which are strongly influenced by microbial metabolism during fermentation. This review examines three major microbial mechanisms involved in antioxidant enhancement in fermented foods: exopolysaccharide (EPS) production, release of matrix-bound bioactive compounds, and microbial modulation of redox conditions. Microbial EPS contribute through radical scavenging and metal chelation, while microbial enzymes increase the bioavailability of phenolic compounds, peptides, and other antioxidant molecules. In addition, microbial metabolic activity influences the redox environment of fermented systems through electron-transfer processes and reducing metabolites. By integrating these complementary mechanisms, this review provides a comprehensive framework linking microbial biotransformation and redox modulation to the antioxidant properties of fermented foods, and highlights their potential for the development of functional fermented products.

## 1. Introduction

Oxygen is an essential molecule for aerobic life. It inevitably leads to the formation of reactive oxygen species (ROS) and reactive nitrogen species (RNS), which can cause significant cellular damage. These highly unstable entities, characterized by the presence of one or more unpaired electrons, are capable of independent existence and can oxidize biological macromolecules [1]. From that, a new field has emerged in dietary strategies, leading to the expansion of the food and supplement industry, which focuses on compounds that can counteract the effects of oxidant molecules and scavenging free radicals [2]. Several studies have shown that ROS and RNS play a crucial role in cell signaling, explaining why, for instance, antioxidant supplements do not always benefit from exercise training [3]. Fermentation is a food-processing method that emerged alongside the development of agriculture. Fermented foods are currently attracting renewed nutritional and scientific interest because their consumption is associated with improved sensory, preservative, and, in some cases, functional properties. During fermentation, microorganisms transform food matrices through diverse metabolic activities. Fermentation is considered a sustainable and natural process that can improve food quality without requiring synthetic additives [4]. Fermenting microorganisms should not be systematically classified as probiotics. Although fermenting microorganisms are essential for the biochemical transformation of food matrices, they should not be systematically classified as probiotics. According to internationally accepted definitions, probiotics are live microorganisms that, when administered in adequate amounts, confer a scientifically demonstrated health benefit on the host. Probiotic status therefore requires strain-specific identification, safety assessment, and clinical validation. Consequently, many microorganisms involved in food fermentation do not meet these criteria, making it important to distinguish fermented foods from probiotic products [5].

Beyond microbial activity itself, fermented foods may contain antimicrobial and physiologically bioactive compounds generated during fermentation. Among these are molecules capable of modulating oxidative processes through antioxidant, radical-scavenging, or synergistic effects [6]. In addition, the consumption of fermented dairy products has been associated with a reduced risk of type 2 diabetes and improved cardiometabolic health [7,8]. For instance, bacteria producing lactic acid from sugars will be inactivated by this acid waste, but, in the meantime, several metabolic mechanisms are used by bacteria to decrease the acidity of the medium [9]. Likely, the production of antioxidant compounds during fermentation is also resulting from a balance between toxic wastes and mechanisms to improve the oxidation conditions in the medium. In that case, contrasting with a supplementation of diets with a high concentration of bioactive molecules, microorganisms would be able to sense the oxidation level in their environment and act carefully to adjust it to living requirements [10].

This review focuses on microbial mechanisms contributing to the antioxidant properties of fermented foods, including exopolysaccharide production, release of bioactive compounds from food matrices, and microbial modulation of redox conditions.

## 2. Microbial Exopolysaccharides and Their Contribution to Antioxidant Activity in Fermented Foods

Microbial exopolysaccharides (EPS) are extracellular carbohydrate polymers produced by bacteria, yeasts, fungi, microalgae, and archaea [11,12]. These polymers have attracted considerable interest because of their technological, biological, and functional properties, including their contribution to the antioxidant potential of fermented foods [13].

Microbial polysaccharides are generally classified into intracellular storage polysaccharides, capsular polysaccharides, and extracellular polysaccharides. Among these, extracellular polysaccharides, commonly referred to as exopolysaccharides (EPS), are secreted into the surrounding environment and include polymers such as xanthan, alginate, cellulose, and dextran [11]. Depending on their monosaccharide composition, EPS are further divided into homopolysaccharides (HoPS), composed of a single type of monosaccharide, and heteropolysaccharides (HePS), which contain two or more different monosaccharides such as glucose, galactose, mannose, or rhamnose [14].

EPS play an important role in microbial physiology and environmental adaptation. They form a hydrated extracellular matrix that contributes to cell adhesion, biofilm formation, structural stability, and protection against environmental stress [15]. Within biofilms, EPS facilitate nutrient diffusion, maintain hydration, and improve microbial tolerance to adverse conditions such as osmotic stress, temperature fluctuations, and desiccation [16]. In addition, EPS can interact with metal ions and reactive oxygen species (ROS), contributing to oxidative stress protection and microbial survival [17].

### 2.1. Exopolysaccharide Biosynthesis

**Figure 1:**
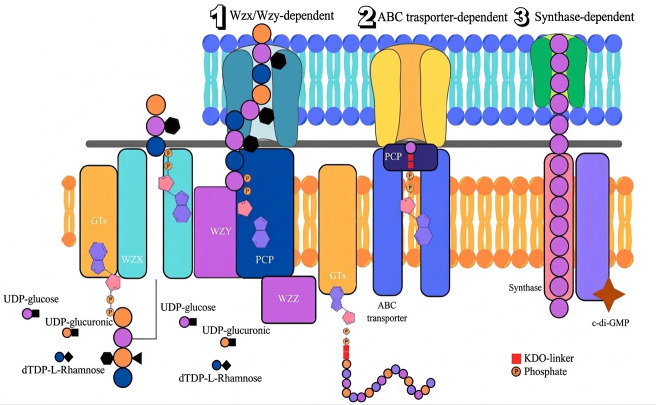
Schematic representation of intracellular EPS biosynthesis pathways. Modified from [11].

Exopolysaccharides (EPS) are synthesized through several intracellular and extracellular pathways (Figure 1), including the Wzx/Wzy-dependent, ABC transporter-dependent, synthase-dependent, and extracellular enzymatic systems [11]. These mechanisms differ in the assembly and export of polysaccharide chains across the cell envelope. In general, homopolysaccharides (HoPS) are mainly synthesized through synthase-dependent or extracellular enzymatic pathways, whereas heteropolysaccharides (HePS) are commonly produced through Wzx/Wzy- or ABC transporter-dependent systems [11].

In the Wzx/Wzy-dependent and ABC transporter-dependent pathways, polysaccharide repeating units are assembled intracellularly by glycosyltransferases before being exported to the cell surface [18,19]. In contrast, the synthase-dependent pathway enables simultaneous polymerization and secretion of the polysaccharide through membrane-associated synthases and is involved in the production of polymers such as alginate and cellulose [20]. Some EPS can also be synthesized extracellularly by cell-associated enzymes such as sucrases that utilize sucrose as a substrate [11].

These biosynthetic pathways generate structurally diverse EPS differing in monosaccharide composition, molecular weight, branching degree, and glycosidic linkages. Such structural diversity strongly influences the physicochemical and biological properties of EPS, including their antioxidant activity.

### 2.2. Exopolysaccharide Antioxidant Potential

Beyond their structural and technological functions, microbial exopolysaccharides (EPS) have attracted considerable interest because of their biological activities, particularly their antioxidant potential [21]. Increasing evidence suggests that EPS contribute to the control of oxidative stress through multiple complementary mechanisms, making them promising functional components in fermented foods and other bioactive systems.

**Figure 2:**
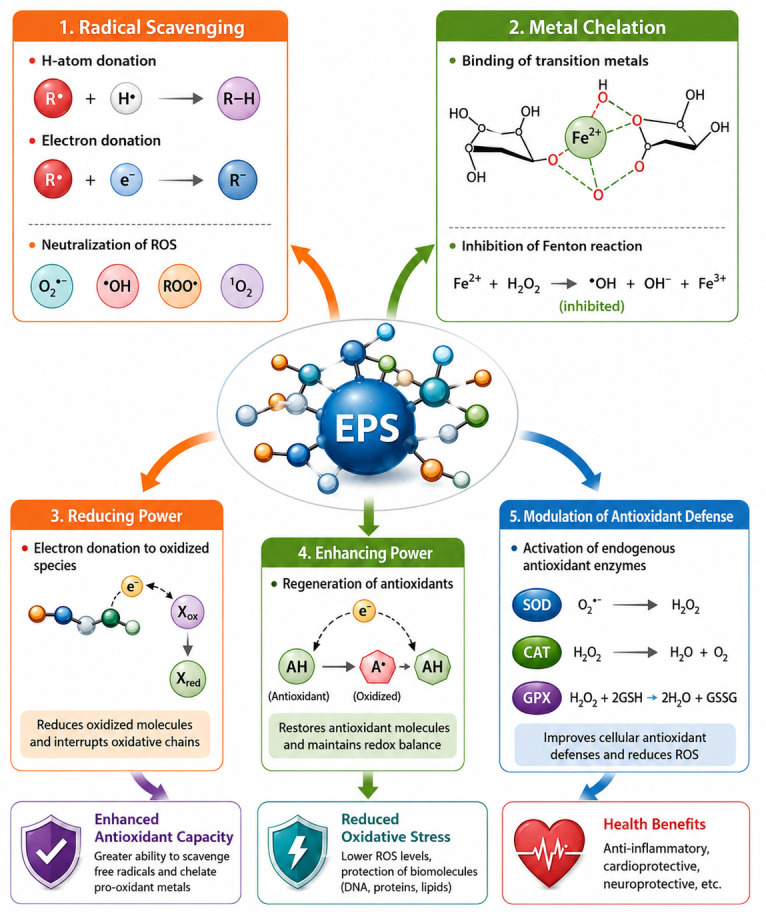
Mechanisms underlying the antioxidant activity of EPS.

Oxidative stress results from an excessive accumulation of reactive oxygen species (ROS), including superoxide radicals, hydroxyl radicals, nitric oxide, and peroxynitrite, which can damage lipids, proteins, and nucleic acids and contribute to the development of chronic diseases [22]. EPS can mitigate oxidative stress through several mechanisms, including radical scavenging, metal ion chelation, reducing activity, and inhibition of lipid peroxidation (Figure 2) [23].

One of the main antioxidant mechanisms of EPS is their ability to neutralize free radicals through hydrogen atom or electron donation. The abundance of hydroxyl groups within polysaccharide chains facilitates interactions with reactive species, leading to the stabilization of free radicals and a reduction in their reactivity [23]. In addition, some EPS can chelate transition metal ions involved in ROS generation, thereby limiting oxidative chain reactions. Other studies have also reported reducing power and modulation of antioxidant defense systems as important contributors to EPS antioxidant activity (Figure 2) [23].

Several microbial EPS have demonstrated significant antioxidant activity in vitro and in vivo (Table 1) [24]. Reported activities include DPPH, ABTS, hydroxyl radical, and superoxide radical scavenging, as well as the enhancement of antioxidant enzymes such as superoxide dismutase (SOD), catalase (CAT), and peroxidases (POD) [25,26,27,28,29,30,31,32,33]. These findings indicate that EPS antioxidant properties depend not only on direct radical neutralization but also on their ability to modulate the oxidative balance in biological systems.

The antioxidant activity of EPS is strongly influenced by their structural characteristics, including monosaccharide composition, molecular weight, glycosidic linkages, branching degree, and chain conformation [34]. EPS rich in glucose residues have shown strong superoxide and DPPH radical scavenging activity, whereas mannose-rich EPS have been associated with an enhanced hydroxyl radical scavenging capacity [35,36]. Molecular weight also appears to influence antioxidant efficiency, although its effect remains context-dependent. While high-molecular-weight EPS may provide improved radical interaction because of their larger structure [37,38], low-molecular-weight fractions have also demonstrated substantial antioxidant potential [39,40].

In addition to monosaccharide composition and molecular weight, structural organization plays an important role in determining antioxidant functionality. Variations in glycosidic linkages and branching degree can affect the electron-donating capacity and interactions with reactive species [39,41]. For example, an α-glucan produced by Bacillus amyloliquefaciens containing α-(1→3) and α-(1→6) glycosidic bonds exhibited superoxide radical scavenging activity [42]. Similarly, highly branched EPS fractions from Tetragenococcus halophilus SNTH-8 showed enhanced antioxidant and emulsifying properties [43].

The conformation of EPS chains may further influence interactions with biomolecules and antioxidant compounds. EPS produced by *Lactiplantibacillus plantarum* have demonstrated both in vitro and in vivo antioxidant effects, partly due to their ability to interact with food proteins such as casein and stabilize antioxidant systems [44]. These observations highlight the multifunctional nature of microbial EPS and emphasize the complex relationship between EPS structure and antioxidant activity [45,46].

Overall, microbial EPS represent important contributors to the antioxidant properties of fermented foods. However, the relationship between EPS structure and antioxidant function remains complex and requires further investigation to better understand their mechanisms of action and optimize their application in food and pharmaceutical systems.

### 2.3. Exopolysaccharides as Contributors to the Antioxidant Potential of Fermented Foods

Several studies have demonstrated that microbial exopolysaccharides (EPS) contribute to the antioxidant properties of a wide range of fermented foods [47]. In dairy products such as yogurt, kefir, and fermented milk, EPS produced by lactic acid bacteria, including *Lactiplantibacillus plantarum*, *Lactococcus lactis*, *Streptococcus thermophilus*, and *Leuconostoc mesenteroides*, have been associated with enhanced antioxidant activity and improved physicochemical properties. For example, EPS produced by *L. plantarum* strains isolated from fermented foods exhibited strong DPPH, hydroxyl radical, and superoxide radical scavenging activities, suggesting their contribution to the antioxidant capacity of fermented dairy products [48].

In fermented food systems, however, antioxidant activity does not rely solely on EPS production (Table 2). Instead, it results from a complex network of interactions involving microbial metabolism, enzymatic transformations, and the release of bioactive compounds from food matrices [49,50]. During fermentation, microorganisms produce enzymes such as β-glucosidases, esterases, and phenolic acid decarboxylases that hydrolyze bound phenolic compounds and increase their bioavailability and antioxidant activity [49]. Proteolytic activity can also generate bioactive peptides with antioxidant properties, further enhancing the functional value of fermented foods [51].

**Figure 3:**
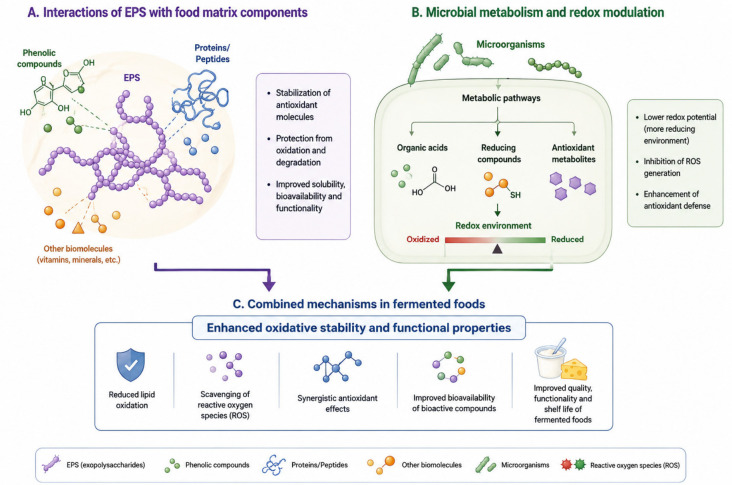
Contribution of EPS to antioxidant properties of fermented foods.

Beyond their direct antioxidant effects, EPS may interact with phenolic compounds, proteins, and other biomolecules within the food matrix, contributing to the stabilization of antioxidant molecules and improving their functionality (Figure 3). In parallel, microbial metabolism can modify the redox environment through the production of organic acids, reducing compounds, and antioxidant metabolites. These combined mechanisms contribute to the oxidative stability and functional properties of fermented foods [50].

Overall, the antioxidant potential of fermented foods arises from synergistic interactions between EPS production, microbial enzymatic activity, and the release or transformation of natural antioxidant compounds during fermentation. This highlights the importance of microbial biotransformation as a key factor in enhancing the functional and antioxidant properties of fermented food systems.

## 3. Release of Matrix Antioxidants Through Hydrolase Activities

The release of antioxidant molecules from plant matrices is a critical area of research, particularly in the context of food science, nutrition, and pharmacology. Glycosidases and esterases play pivotal roles in the hydrolysis of glycosidic bonds and ester linkages, respectively, facilitating the bioavailability of phenolic compounds, flavonoids, and other antioxidants. This review explores the mechanisms by which these enzymes contribute to the release of antioxidant molecules, the implications for health benefits, and potential applications in food processing and nutraceutical development.

Fermentation provides a biotechnological approach to improve plant antioxidant bioaccessibility by releasing antioxidants from plant matrix glycosides and complexes [52]. In addition to increasing the concentration of antioxidant compounds, fermenting changes their molecular structure, increasing their solubility and bioavailability, which makes them more effective in the human body [53].

Verni et al. suggest that fermentation releases or converts these compounds into active forms and attribute these changes to phenolic, flavonoid and tannic metabolic activity, antioxidant peptide release, vitamin changes and exopolysaccharide production [54].

Microbial hydrolysis, which leads to an increase in the amount of phenolic compounds and flavonoids in the food, has been suggested as the main reason for the positive correlation between fermentation and antioxidant activity. Furthermore, the structural breakdown of plant cell walls leads to the release or synthesis of the various antioxidant compounds mentioned above [55]. These compounds can act as scavengers of free radicals, chelators of metals, inhibitors of singlet oxygen, or hydrogen donors to free radicals [49].

Some authors [56] proposed that polyphenols have the capacity to promote the growth of probiotic bacteria whilst concomitantly inhibiting the growth of harmful bacteria. The term ‘duplibiotic’ has been proposed to describe this dual action of polyphenols [57]. Polyphenols have also been shown to stimulate the secretion of polyphenol-related enzymes (tannase, gallate decarboxylase, esterase, phenolic acid decarboxylase and glycosidase) by intestinal microorganisms, facilitating the metabolism of difficult-to-digest phenols into nutritional growth factors [58]. Furthermore, microorganisms interact with polyphenols during the process of food fermentation, resulting in the modification of the quality of fermented foods under the dual action of polyphenols. Concurrently, the hydrolysis of polyphenols by micro-organisms has the potential to enhance the bioavailability of polyphenols in food materials, thereby producing a series of bioactive components that are more conducive to digestion, absorption, and human utilization [58].

**Figure 4:**
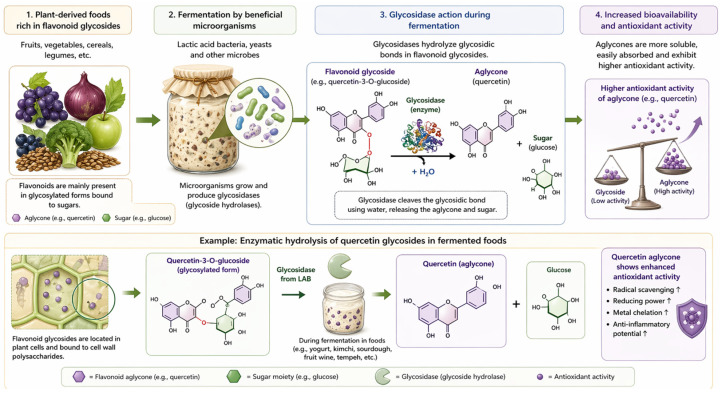
Glycosidase-mediated hydrolysis of flavonoid glycosides and enhancement of antioxidant activity in fermented foods.

Glycosidases, also known as glycoside hydrolases, catalyze the hydrolysis of glycosidic bonds in glycosides, releasing aglycones that often possess higher antioxidant activity (Figure 4). For instance, flavonoid glycosides, which are prevalent in fruits and vegetables, can be converted into their aglycone forms by glycosidases, enhancing their antioxidant capacity [59]. Quercetin, a well-known flavonoid, is often found in glycosylated forms in various plant sources. Studies have shown that the enzymatic hydrolysis of quercetin glycosides by specific glycosidases significantly increases the antioxidant activity of the released aglycone [60].

**Figure 5:**
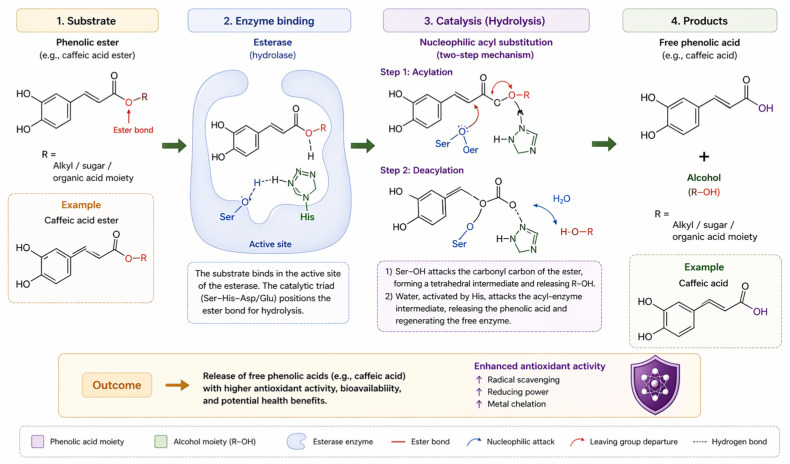
Esterase-mediated hydrolysis of phenolic esters and release of antioxidant phenolic acids in fermented foods.

Esterases catalyze the hydrolysis of ester bonds, which can also lead to the release of antioxidant compounds. For example, phenolic esters found in plant matrices can be hydrolyzed by esterases, resulting in the liberation of free phenolic acids that exhibit strong antioxidant properties [61]. Caffeic acid, a potent antioxidant, is often present in esterified forms in various plant tissues (Figure 5). The action of esterases can release caffeic acid, thereby enhancing its bioavailability and antioxidant potential [61].

Several factors can influence the activity of glycosidases and esterases [62], including:

1. pH and temperature: Optimal pH and temperature conditions are crucial for maximizing enzyme activity. For instance, many glycosidases exhibit peak activity at neutral pH and moderate temperatures.
2. Substrate specificity: Different enzymes have varying substrate specificities, which can affect the efficiency of antioxidant release. The choice of enzyme is critical for targeted applications.
3. Matrix composition: The presence of other compounds in plant matrices, such as proteins and polysaccharides, can inhibit or enhance enzyme activity.

The enzymatic release of antioxidants has significant implications for food processing and the development of functional foods. By employing glycosidases and esterases, food manufacturers can enhance the antioxidant content of products, improving their health benefits. For example, the use of these enzymes in fruit juices can increase the bioavailability of antioxidants, making them more effective in combating oxidative stress [63].

## 4. Microbial Modulation of Oxidative Process in Fermented Food

In addition to producing antioxidant compounds and releasing bioactive molecules from food matrices, fermenting microorganisms can influence the redox environment of fermented foods through their metabolic activities. These processes contribute to the modulation of oxidative reactions and may indirectly enhance the antioxidant stability of fermented products.

During fermentation, microorganisms generate various metabolites, including organic acids, reducing compounds, and bioactive peptides, that can interact with reactive oxygen species (ROS) and influence global redox balance. Some microorganisms also produce antioxidant enzymes such as superoxide dismutase and catalase, which contribute to protection against oxidative stress [54,64,65]. Among fermenting microorganisms, lactic acid bacteria (LAB) have been extensively studied because of their strong capacity to modify the redox conditions of fermented systems [66,67,68,69].

LAB are generally considered anaerobic or oxygen-tolerant microorganisms with predominantly reducing metabolism. Some species can also perform respiration in the presence of suitable electron acceptors, improving their tolerance to oxidative stress and influencing redox conditions during fermentation [70,71,72]. Beyond metabolic activity, microbial surface properties may also contribute to redox regulation. Studies on LAB have shown considerable variability in electron donor and acceptor capacities, which may affect microbial interactions, oxidative balance, and adaptation within fermented food matrices [73,74,75,76,77,78,79,80,81].

Several studies have demonstrated that LAB can actively reduce the redox potential of culture media and fermented foods [67,69,82,83,84,85,86,87,88,89,90,91]. Species such as *Lactococcus lactis* and *Lactiplantibacillus plantarum* exhibit particularly strong reducing capacities [69,91]. In complex fermented systems, microbial metabolism and oxygen distribution can generate localized redox gradients that influence microbial activity and the stability of antioxidant compounds [66].

The reduction in redox potential in fermented systems has been associated with multiple microbial factors, including the presence of surface-exposed thiol groups that can participate in electron-transfer reactions [92]. These observations suggest that microbial surface properties, together with microbial metabolism, contribute to oxidative balance and ecological adaptation in fermented environments. Overall, microbial modulation of redox conditions represents an important complementary mechanism contributing to the antioxidant properties and functional stability of fermented foods.

## 5. Current Challenges and Future Perspectives on Microbial Exopolysaccharides as Antioxidants in Fermented Foods

Despite the great interest in EPS as natural antioxidants, several limitations still hinder a comprehensive understanding of their role in fermented foods. One of the biggest problems is that microbial EPS have a very wide range of structures. Differences in molecular weight, monosaccharide composition, glycosidic linkage configuration, and degree of branching can significantly influence antioxidant activity, but clear structure–activity relationships remain difficult to establish. Moreover, the majority of studies assessing the antioxidant capacity of EPS are performed in vitro (e.g., DPPH, ABTS, or hydroxyl radical scavenging tests) [93,94,95]. These assays yield valuable preliminary data; however, they inadequately capture the intricate nature of oxidative processes in food matrices and biological systems. Consequently, the biological significance of numerous documented antioxidant effects necessitates additional validation through cell-based models, gastrointestinal digestion simulations, and in vivo investigations.

Interactions between EPS and other food components, like proteins, lipids, and polyphenols, are not completely understood. EPS can bind to proteins or phenolic compounds, which may change the way they are structured and how they work in the body. Furthermore, the stability of EPS during food processing, storage, and gastrointestinal digestion is inadequately understood, and these factors may substantially affect their ultimate functional impact in the human body [96].

From a practical point of view, choosing and improving EPS-producing starter cultures is a good way to make fermented foods better in both their technological and functional aspects. Progress in microbial genomics and metabolic engineering is creating new chances to find strains with better EPS biosynthetic pathways and useful functional properties. Fermentation conditions like temperature, pH, substrate composition, and microbial interactions can also have a big effect on EPS yield and structure. It will be very important to know how these things affect EPS production in order to make fermentation processes that make the best products and have health benefits [97].

Consequently, forthcoming research should concentrate on several pivotal domains. First, a more thorough structural characterization of microbial EPS is necessary to elucidate the correlation between chemical structure and antioxidant activity, employing advanced analytical methodologies such as nuclear magnetic resonance (NMR), mass spectrometry, and chromatographic techniques. Second, the assessment of EPS bioactivity should progressively integrate physiologically pertinent models, encompassing cell cultures, animal models, and human studies. Third, greater attention should be given to the interactions between EPS and other components of fermented foods. Finally, integrating microbiological, biochemical, and food technology approaches will be essential to fully exploit microbial EPS as natural antioxidants and to support the development of innovative functional fermented foods with potential health benefits.

## 6. Conclusions

From this study, it seems that the antioxidant properties of fermented food are not only related to a coincidental production of highly antioxidant compounds, which could come for instance from glycosylated phenolic compounds from a plant matrix, but may be more related to a microbial activity of cells aiming at decreasing oxidation threats and/or managing redox conditions supporting metabolic goals. In this field, we can cite the recent study about cheese-LAB persistence in the mouth, which seems driven by the oxidation state of the oral cavity [98]. Although the driving of the matrix redox potential by bacteria, which has been characterized for its biotechnological interest [91], has still to be studied in its ecological dimension, many physiological signs show that electron active molecules, like exofacial thiol groups [92], are produced in reaction to the environment. It is interesting to note that the EPS structure, which protects microorganisms in many conditions, exhibits strong antioxidant properties [99].

**Figure 6:**
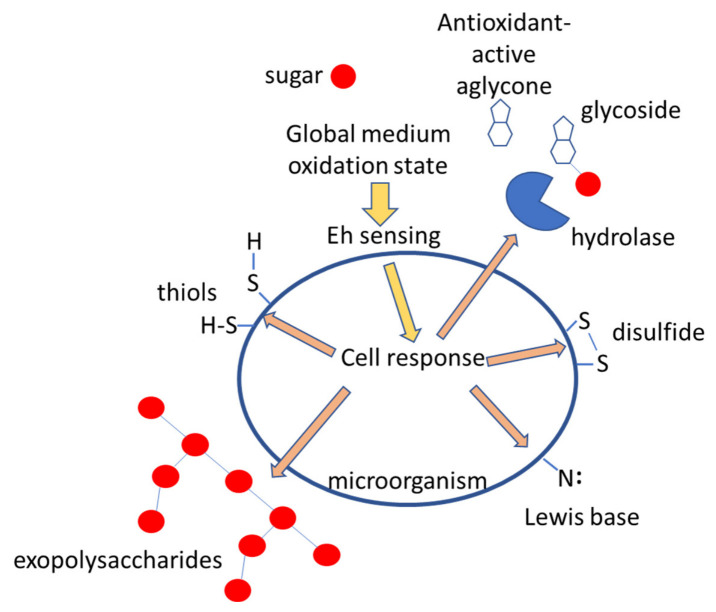
Scheme showing the various impacts of microorganisms on the oxidation/antioxidation state of their environment.

Taken together and as illustrated in Figure 6, these elements suggest that, in fermented foods, microorganisms are correcting oxidation excesses, making these foods healthier from an oxidation or antioxidant perspective.

## Figures and Tables

**Table 1:** Monosaccharide compositions of EPS with antioxidant activity. Modified from [24].

EPS-Producing Microorganism	Monosaccharide Composition	Molecular Weight	Antioxidant Assays	Proposed Antioxidant Mechanism	Reference
*Pantoea alhagi* NX-11	Glucose, galactose, mannose	1.326 × 10^6^ Da	Plant antioxidant enzyme activity (SOD, CAT, POD); MDA reduction under salt stress	Induction of antioxidant defense system and ROS mitigation in plants	[25]
*Lysinibacillus fusiformis* KMNTT-10	Xylose, rhamnose, arabinose, galactose, glucose	n.r.	DPPH, ABTS and nitric oxide radical scavenging	Direct free radical scavenging and NO neutralization	[26]
*Leuconostoc mesenteroides* LM187	Arabinose, galactose, rhamnose	7.757 × 10^7^ Da	DPPH, hydroxyl radical and superoxide radical scavenging; reducing power	Hydrogen atom donation and electron transfer capacity	[27]
*Weissella confusa* XG-3	Glucose	3.19 × 10^6^ Da	DPPH, hydroxyl radical and superoxide radical scavenging	ROS scavenging and electron-donating capacity	[28]
*Weissella cibaria* MED17	Glucose (α-glucan)	n.r.	DPPH, ABTS and hydroxyl radical scavenging; reducing power	Hydrogen atom donation and radical neutralization	[29]
*Lactiplantibacillus plantarum* HY	Mannose, galactose, glucose	9.549 × 10^4^ Da	DPPH, hydroxyl radical and superoxide radical scavenging	Direct ROS scavenging and reducing capacity	[30]
*Abortiporus biennis*	Glucose, mannose, galactose	2.207 × 10^4^ Da	DPPH radical scavenging; reducing power	Electron donation and radical neutralization	[31]
*Lactiplantibacillus plantarum* JLAU103	Arabinose, rhamnose, fucose, xylose, mannose, fructose, galactose, glucose	1.24 × 10^4^ kDa	DPPH, hydroxyl radical and superoxide radical scavenging	Radical scavenging and possible metal ion chelation	[32]
*Limosilactobacillus fermentum* S1	Mannose, rhamnose, glucose, galactose	7.19 × 10^5^ Da	DPPH, hydroxyl radical and superoxide radical scavenging; reducing power; in vivo antioxidant activity	ROS scavenging and enhancement of antioxidant defense enzymes	[33]

n.r = Not Reported.

**Table 2:** Main microbial mechanisms contributing to antioxidant and redox modulation in fermented foods.

Mechanism	Main Microbial Activity	Key Molecules/Processes Involved	Contribution to Antioxidant Activity	Examples of Fermented Foods
Microbial exopolysaccharides (EPS)	Production of extracellular polysaccharides by fermenting microorganisms	Radical scavenging, metal chelation, reducing power, stabilization of antioxidant compounds	Neutralization of ROS, protection against oxidative degradation, improvement of oxidative stability	Yogurt, kefir, fermented milk, sourdough
Enzymatic release and transformation of bioactive compounds	Microbial hydrolysis and biotransformation of food matrix components	β-glucosidases, esterases, phenolic acid decarboxylases, proteases	Release of phenolic compounds, generation of antioxidant peptides, increased bioavailability of antioxidants	Fermented cereals, vegetables, dairy products
Microbial modulation of redox balance	Metabolic and electron-transfer activities influencing redox conditions	Organic acids, reducing metabolites, antioxidant enzymes, thiol groups, electron transfer systems	Lowering redox potential, stabilization of antioxidant molecules, limitation of oxidative reactions	LAB-fermented dairy products, vegetable fermentations

## Data Availability

No new data were created or analyzed in this study. Data sharing is not applicable to this article.
